# Surgical Complications and Its Grading: A Literature Review

**DOI:** 10.7759/cureus.24963

**Published:** 2022-05-13

**Authors:** Roshani S Manekk, Pankaj Gharde, Rajesh Gattani, Yashwant Lamture

**Affiliations:** 1 Department of General Surgery, Datta Meghe Institute of Medical Sciences, Wardha, IND

**Keywords:** negative outcomes, grading, classification, abdominal surgery, complications

## Abstract

The demand for improvement in healthcare delivery has been increasing. Thus, a standardized method allows quality assessment of data and its comparison between various institutions over time. Many attempts have been made to classify surgical complications before 1990; however, none of those attempts gained popularity and acceptance. Clavien and his colleagues started the wave by explaining negative outcomes on the basis of complications, failure to cure, and sequelae. Complications were primarily defined as “any deviation from the normal postoperative course”. Since then, many such classification systems and grading systems have been introduced and studied for analyzing the post-operative complications. The purpose of this study was to review the revolution in the classification systems for surgical complications, its validation, and to analyze the results of various qualitative indicators for post-operative complications obtained by using these classification systems. A global set of keywords were built such as “grading of surgical complications”, “abdominal surgery”, “classification of surgical complications”, and the “Clavien Dindo Classification”. A literature review was done using PubMed, Medline, and Google Scholar. A list of reference articles concerning the literature on classification systems for surgical complications was manually analyzed from the year 1992 and the data was summarized.

## Introduction and background

Despite good surgical techniques and the presence of skilled surgeons, post-surgical complications have always been the most difficult part of the management of patients [1]. The demand for improvement in healthcare delivery has been increasing; thus, a standardized method allows quality assessment of data and its comparison between various institutions over time [2]. Many attempts have been made to classify surgical complications before 1990; however, none of those attempts gained popularity and acceptance. A standardized method of classification of surgical complications was proposed by Clavien et al. in 1992 which is known as the T92 system or Clavien classification of surgical complications [2]. In 2004, Clavien along with Dindo revised the basic T92 model which was later named as “Clavien-Dindo Classification”. The authors studied and provided evidence of five years of experience for the proposed classification. Martin et al. made minor modifications to the Clavien-Dindo Classification (CDC) system, which came to be known as the Memorial Sloan Kettering (MSKCC) Severity Grading System [3]. The Accordion Severity Grading System of surgical complications was described by Strasberg et al. in 2009. The grading system is complex in nature and can expand the range of complications in complex studies. The contracted classification had four levels, whereas the expanded classification had six levels. The proposed time horizon for recording complications was extended to 100 days after the surgical procedure [4]. The comprehensive complication index (CCI) was defined in 2013 by Slankamenac et al. [5]. The authors focused on the criteria that the Clavien-Dindo classification system graded as the single most severe complication that occurred in the patient, thus ignoring the less severe events. This fails to represent the true overall “morbidity”, after surgery. The authors thus adopted the “operation risk index” approach for developing the mathematical formula of CCI. They combined the complications according to the severity into single score from 0 to 100. This helps to measure a cluster of complications at a given period of time [5]. In 2015, the “Japan Clinical Oncology Group” (JCOG) aimed to standardize the terms for defining adverse events (AE) as per the Clavien-Dindo Classification system. The criteria were defined based on extensive research, done by nine surgical specialties, in which they specified the complications commonly experienced in their field [6]. The purpose of this study was to review the revolution in the classification systems for surgical complications, its validation, and to analyze the results of various qualitative indicators for post-operative complications obtained by using these classification systems.

Methods

**Figure 1:**
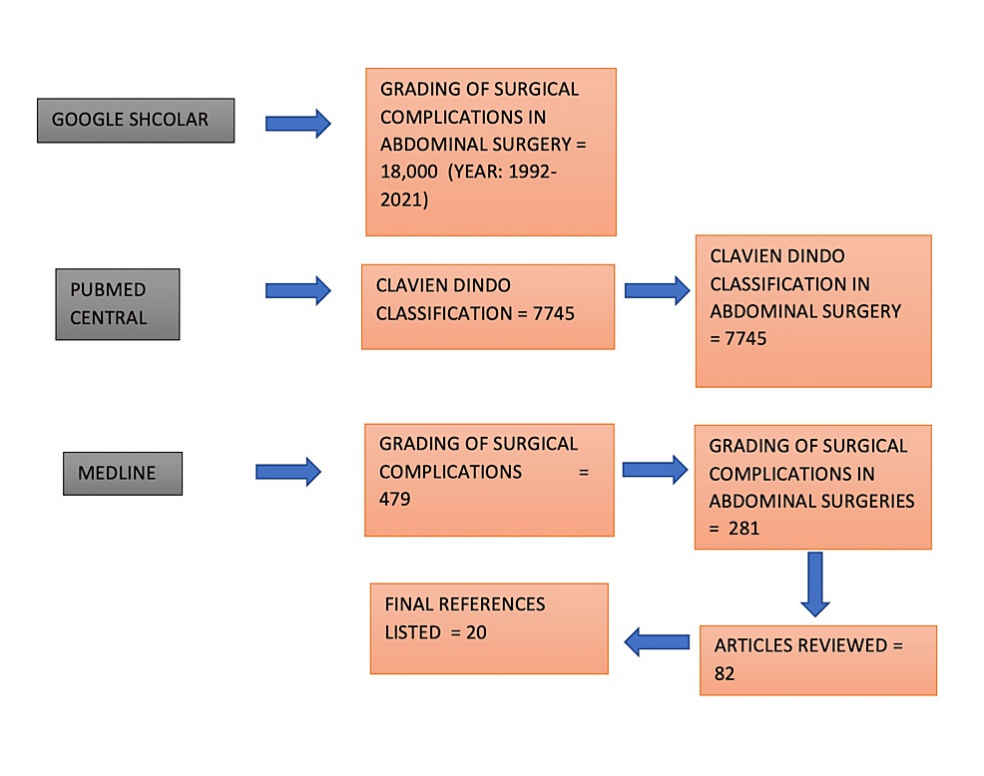
Schematic Representation of Methods for Data Collection.

As per PRISMA guidelines, the topic for the literature review was selected for the purpose of summarizing various classification systems. This was done using PubMed, Medline and Google Scholar search engines. A set of keywords were used for the purpose of data collection like, “grading of surgical complications”, “abdominal surgery”, “classification of surgical complications”, and the “Clavien Dindo Classification”. Level I to level IV evidence-based reference articles concerning the literature on the classification systems for surgical complications were manually analyzed from the year 1992. A total of 82 articles were studied and finally, 20 references were used for the current literature as shown in Figure 1.

## Review

Surgical complications

As per the literature, the incidence of post-operative complications remains one of the most commonly used parameters, to mark the quality of surgery. However, the definition of surgical complications (SC) lacks a standard definition [7].

Starting in 1992, Clavien and his colleagues started the wave by explaining ‘negative outcome’ on the basis of complications, failure to cure and sequelae. The primary definition of complications was “any deviation from the normal postoperative course”. A sequela is defined as an “after-effect” of surgery that is inherent to the procedure (For example, a person’s inability to be able to walk after undergoing amputation). Finally, a surgery can fail to serve its original purpose. This is described as a failure to cure (For example, presence of residual tumor after resection) [2]. In 2004, the same authors proposed a new classification system to grade the surgical complications. The concepts of sequelae and failure to cure were not included while forming this classification [7].

Various definitions for complication

Veen et al. (1999) in the “European Journal of Surgery” defined complications as “every unwanted development in the illness of the patient or in the treatment of patient’s illness that occurs in the clinic”. This classification is also known as the T92 (Toronto 1992) complication grading system [8]. In 2007, a well-known historian in science proposed that “a complication, in any sphere of an endeavour is something out of the norm and the product of extraneous and unexpected factors”. Sokol and Wilson in a study defined SC as, “an undesirable, unintended and direct result of an operation affecting the patient which would not have occurred had the operation gone as well as could reasonably be hoped”. The basis of the definition of 2008 as thought by authors was that an SC is not a fixed entity, and is dependent on the surgical skills acquired by the professional, and also the facilities available, i.e., the SC in the United Kingdom (UK) may not count as an SC in rural India. Similarly, a complication might lose its value over time [9]. This definition was reviewed by the authors of CD classification in the same year, and the original definition given by Clavien and Dindo was modified to “any deviation from the ideal postoperative course that is not inherent to the procedure, and does not comprise failure to cure” [7].

Evolution of classification for grading surgical complications

The initial classification system proposed by Clavien in 1992 is known as “The Clavien Classification System (CCS)” which emphasizes on morbidity and the therapeutic treatment used for complications, and for determination of the severity of complications. This system is also known as “The T92 Classification System” [10]. This classification is comprised of four grades of severity as shown in Table 1.

**Table 1:** The Initial T92 Classification System.

Grade	Definition
Grade I	Any complication which would resolve spontaneously if left untreated without the need for pharmacological intervention. Hospital stay required for treatment of complication does not exceed twice the median length of stay for the procedure.
Grade II	Potentially life-threatening complication with the need for some form of intervention. Does not result in lasting or residual disability or organ resection.
Grade IIa	Complications requiring medications other than allowed for Grade I.
Grade IIb	Complications requiring invasive procedures or reoperation.
Grade III	Complications with residual or lasting disability or which require organ resection.
Grade IV	Death as a result of any complication.
Note - Medications in Grade I complications include: analgesic, antipyretic, antiemetic and antidiarrheal drugs.	

Clavien along with Dindo proposed a revised model of the same CCS, and later named it as Revised Clavien Dindo Classification 2004. Two subgroups were added in Grade III and Grade IV. Grades I and IIa of the CCS correspond to Grade II of CD classification. The Grade IIb events in CCS were now listed as a separate Grade III in the CD classification. Grade IIIb was further subdivided into groups IIIa and IIIb based on the type of anesthesia used. The length of hospital stay (LOS) criteria used to rank Grade II complications in CCS were eliminated. Potentially life-threatening complications initially defined in Grade II of CCS were now moved to a higher grade in CD classification, i.e., Grade IV. Disability, a criterion for Grade III CCS, is now considered as a separate entity in CD classification. It is highlighted by using a suffix “d”, and can be added to any grade. Disability is defined by authors as “any impairment of body function” [2]. This classification system was re-evaluated in 2009 by the pioneers, using complicated clinical scenarios from the University of Zurich weekly morbidity, and mortality (M and M) conferences. Surgeons from seven centers across the world graded these complications with >90% agreement [11]. The Accordion Severity Grading System 2009 has the ability to grade a wide range of post-operative complications. The contracted version of this classification system has four levels, and the expanded version has six levels. The expanded classification system is used for complex procedures like pancreatic or esophageal resection. The major difference between the two types is the expansion of the severe group into three subgroups, as shown in Table 2.

**Table 2:** Accordion Severity Classification of Postoperative Complications: Contracted and Expanded.

Contracted Classification	Expanded Classification
1. Mild complication: Minor invasive procedures, done at the bedside. Physiotherapy and the following drugs are allowed: antiemetics, antipyretics, analgesics, diuretics, electrolytes, and physiotherapy.	1. Mild complication: Minor invasive procedures done at the bedside. Physiotherapy and the following drugs are allowed: antiemetics, antipyretics, analgesics, diuretics, electrolytes, and physiotherapy.
2. Moderate complication: Treatment with drugs other than such allowed for minor complications, for example, antibiotics. Blood transfusions and total parenteral nutrition are also included.	2. Moderate complication: Treatment with drugs other than such allowed for minor complications, for instance, antibiotics. Blood transfusions and total parenteral nutrition are also included.
3. Severe complication: Complications requiring endoscopic or interventional radiologic procedures or re-operation as well as complications resulting in failure of one or more organ systems.	3. Severe: Management by an endoscopic, interventional procedure or re-operation without general anesthesia.
4. Death: Postoperative death.	4. Severe: Management under general anesthesia.
	5. Severe: Organ system failure
	6. Death: Postoperative death.

These categories were made on the basis of levels IIIA, IIIB, IVA and IVB of the CD classification. The authors have defined the terminology, organ system failure, and refer to the new-onset organ failure in postoperative period as shown in Table 3 [4].

**Table 3:** Definition of Organ Failure as per Accordion Classification of Postoperative Complications. CNS- Central Nervous System; GCS- Glasgow Coma Scale; FFP- Fresh Frozen Plasma; INR- International Normalized Ratio; SOFA Score- Sequential Organ Failure Assessment Score.

Cardiac	Need for any of following medications: Epinephrine (>0.1g/kg/min), Norepinephrine (>0.1g/kg/min) or Dopamine (>15g/kg/min)
CNS	GCS less than or equal to 6
Hematologic	Platelet count less than 20 x 10^9^/L
Liver	Need for FFP to correct INR in patient with serum bilirubin >12 mg/dL (205 mMoles/L) OR INR >2.5 in patient with serum bilirubin >12 mg/dL (205 mMoles/L)
Renal	Need for dialysis in patient not on dialysis preoperatively
Respiratory	Need for mechanical ventilation for greater than 24 h in a patient who requires reintubation after surgery OR need for mechanical ventilation of greater than 72 h in a patient who is not extubated on the day of surgery. Does not include patients already on a mechanical ventilator for respiratory failure.
Note- The definitions used here for failure in cardiac, CNS, and hematologic systems are derived from definitions of “score 4” in the SOFA scale. The definition for liver failure is derived in part from the SOFA scale, which uses bilirubin >12 mg/dL as the sole criterion. The definitions for Renal and Respiratory failure rely on the need for dialysis and mechanical ventilation in keeping with the basic concept of T92 that the severity is reflected by the treatment.	

Differences between “the CD classification system and the Accordion Severity Grading System”

As per the CD classification, Grade IV complications like “life-threatening complications including CNS complications which require ICU management are subdivided into single and multiorgan failure (MOF), and are classified as Grade IVA and IVB, respectively. The Accordion Severity Grading System has removed ICU admission requirement criterion, and the classification now includes, only the presence of organ system failure as criteria.

The comprehensive complication index

In 2019, validation of this index was carried out by Park et al. in a small volume hospital in Taiwan. The results of this study revealed that the index was well described in patients with a higher grade of Clavien-Dindo classification. The comprehensive complication index (CCI) reflected any complication status with better distribution when compared with the Clavien-Dindo classification [12]. Various studies showing the significance of CCI with different variables like pain scale, cognitive function, and length of hospital stay (LOS) have been shown in Table 4.

**Table 4:** Studies Showing the Significance of Comprehensive Complication Index (CCI) With Variables.

Variables	Significance of CCI
Pain scale	Significant (Park et al.) [12], p = 0.037
Cognitive function scale	Significant (Park et al.) [12], p = 0.048
Pre-operative Charlson comorbidity index	No statistical significance (Park et al.) [12]
Length of hospital stay (LOS)	Significant (Tirotta et al.) [13], p < 0.001

Tirotta et al. compared both classifications in patients undergoing primary retroperitoneal sarcoma surgery. They reported that, even though, both the classification systems were correlated with LOS, the association with CCI was more significant [13]. A similar result was given by Kim et al. in a study done on radical gastric cancer surgery patients. The authors had an opinion that the CCI was superior to the Clavien-Dindo classification system. Since the Clavien-Dindo classification records only the highest grade of complications, it produces incomplete data as compared to CCI. They report that the use of CCI can be helpful for monitoring the performances of different surgeons as well as it can help to monitor the surgical outcomes at an institutional level [14]. A study done by Veličković et al. in Serbia also reports that the CCI and the Clavien-Dindo classification both are very useful methods for reporting outcomes of complications after major abdominal surgery. However, the CCI has proven to be better in accuracy for recording complications in high-risk patients. They also report that CCI correlated better with LOS when compared to CDC [15].

Extended Clavien-Dindo classification

In 2015 “Japan Clinical Oncology Group” (JCOG) commissioned members from nine surgical specialities, and defined detailed criteria for grading each complication with respect to the general rules of the CD classification. The JCOG post-operative criteria (JCOG PC) contained 72 surgical adverse events commonly experienced in surgical trials [6].

The analysis of different studies that have used the CD Classification has been shown in Table 5.

**Table 5:** Analysis of Different Studies That Have Used CD Classification.

Study Name	Comment on CD Classification	Other Remarks
Singh et al., 2016 [16]	The CD classification can be used to evaluate the severity of postoperative complications after gastrointestinal perforations.	The majority of the complications were wound infections followed by respiratory complications, burst abdomen, leak and septicemia. The overall mortality (Grade V) in this study was 10.85%. A very high mortality rate was seen in ileal perforation.
Lian et al., 2020 [17]	CD classification plays an essential role, in evaluating post-operative complications in gastric cancer patients.	They concluded that laparoscopic radical gastrectomy is safe and easier with a promising minimally invasive effect in treating gastric cancer and in context to the low incidence of overall complications.
Ma et al., 2021 [18]	Nil	The authors concluded that preoperative comorbidity, age, open surgery and blood loss were independent risk factors associated with early complications following radical gastrectomy. The 5‑year Overall Survival of patients in the severe compli­cation group was worse than those of the non‑severe complication group patients.
Bolliger et al., 2018 [19]	The authors concluded that even in presence of several classifications and clinical scores for the classification of surgical complications, Clavien-Dindo Classification had proven to be an easy, comparable and standard tool in quality management.	Patients who have undergone more complex surgery or those having higher scores were more likely to experience significantly longer lengths of hospital stay.
Wang et al., 2018 [20]	The CD classification system can be broadly applicable with a feasible approach to evaluating Post Pancreatico-duodenectomy Complications (PPCs) in patients following Pancreatico-duodenectomy.	Results showed that preoperative hypoproteinemia could be correlated with all three subdivisions of complications in the study; obstructive jaundice could be associated with only severe PPCs and mortality and older age proved to be an inde­pendent risk factor for mortality.

## Conclusions

It will be justified to conclude that every classification system has its own advantages and disadvantages. The CD classification system is the simplest form of classification that can be used for classifying surgical complications and it is also easily understood by medical as well as paramedical staff. CCI is a more complex index, and should be used for large volume studies for extensive and detailed research on individual postsurgical complications. While CD classification provides only a gross structural idea of the nature of surgical complications, CCI has the advantage of weighing individual complications responsible for a patient’s overall post-surgical outcome.

Length of hospital stay is by far recorded as a significant independent variable for identifying morbidity risk in patients undergoing abdominal surgery. However, an analytic review on the outcome of individual variables on post-operative outcomes will provide us with a better understanding on this subject.
